# Neutrophil activation by *Campylobacter concisus*

**DOI:** 10.1186/1757-4749-5-17

**Published:** 2013-07-03

**Authors:** Nina B Sørensen, Hans L Nielsen, Kim Varming, Henrik Nielsen

**Affiliations:** 1 Department of Infectious Diseases, Aalborg University Hospital, P O Box 365, DK9100 Aalborg, Denmark; 2 Department of Clinical Microbiology, Aalborg University Hospital, P O Box 365, DK9100 Aalborg, Denmark; 3 Department of Clinical Immunology, Aalborg University Hospital, P O Box 365, DK9100 Aalborg, Denmark

**Keywords:** Campylobacter concisus, Neutrophil, Oxidative Burst, CD11b, Inflammation

## Abstract

**Background:**

*Campylobacter concisus* is an emerging enteric pathogen associated with prolonged diarrhoea and possibly inflammatory bowel disease in children as well as adults, but the interaction with cells of the innate immune system is unclear. The magnitude of systemic immunoglobulin response in acute infection is unknown.

**Methods:**

Neutrophils from healthy volunteers were activated with five faecal isolates of *C. concisus* from patients with gastroenteritis as well as the oral reference strain *C. concisus* ATCC33237. Neutrophils were tested for the expression of adherence molecule CD11b by immunoflourescence and for oxidative burst response by chemiluminescence. The opsonic activity in a chemiluminescence assay was assessed with heat treated serum from patients with *C. concisus* infection.

**Results:**

A strong and dose-dependent activation of neutrophil adherence molecule CD11b and oxidative burst response was demonstrated with all six *C. concisus* isolates. Bacteria opsonised in heat treated serum induced an increased chemiluminescence response. Heat treated serum from patients with *C. concisus* infection did not have a higher opsonic activity than heat treated serum from healthy volunteers.

**Conclusion:**

*C. concisus* has the capability to activate the innate immune system by stimulating neutrophil cells to increased adherence molecule expression and oxidative burst response, both crucial for acute inflammation. In a chemiluminescence assay the opsonic activity of heat treated serum from patients was not increased compared to heat treated control serum suggesting a weak systemic IgG response to infection.

## Background

Mucosal inflammation associated with *Campylobacter jejuni/coli* enteritis is well described and *in vitro* examinations of *C. jejuni/coli* have documented activation of neutrophil cells in various assays relevant for acute inflammation [1-5]. Recently another species, *Campylobacter concisus,* has been reported as an emerging enteric pathogen of unclear potential for gastrointestinal disease. In patients with prolonged diarrhoea [6] as well as inflammatory bowel disease [7-9]*C. concisus* is associated with clinical manifestations of mucosal inflammation. However, the mechanisms of action are unknown and the magnitude of host inflammatory response to the pathogen is not well described. Our group previously documented that both faecal and oral isolates of *C. concisus* exhibit the potential to induce impairment of the intestinal barrier function by inducing apoptotic leaks [10]. Furthermore *C. concisus* isolates have been shown to have the ability to attach to and invade human intestinal epithelial cells and induce cytokine production [11]. Proteins of *C. concisus* from Crohn’s disease patients have been identified to be immunoreactive [12] but further knowledge of the human immune reaction towards *C. concisus* is limited.

Neutrophil cells are crucial part of the innate immune system and responsible for immediate local inflammatory responses. Initial and early response of the peripheral blood neutrophil upon stimulation with soluble substances including cytokines is the up-regulation of adherence molecules. When neutrophils subsequently interact with microorganisms, either by phagocytosis or by binding soluble pathogen products at the membrane, an activation of the oxidative metabolism takes place. This is part of the host defence against the microorganisms but is at the same time responsible for tissue changes and features of inflammation. The aim of our study was to examine the capability of clinical isolates of *C concisus* to activate neutrophil cells for pro-inflammatory functions reflecting initial adherence to the endothelium and subsequent oxidative burst response upon activation. Furthermore, we tested the immunoglobulin-dependent opsonic capacity of serum for magnifying the neutrophil response to bacterial challenge and compared serum from patients with culture-proven *C. concisus* infection with serum from healthy controls.

## Results

### Up-regulation of CD11b

Experiments of adherence molecule CD11b up-regulation were carried out with neutrophil cells from three different healthy donors. For all three donors a dose–response pattern was shown towards each of the six isolates investigated. A rapid up-regulation of CD11b occurred at 0.75 CFU/neutrophil (median ratio 8.0; range 7.9-11.0) indicating a quick and substantial response in the early stages of the neutrophil activation. The median CD11b up-regulation ratios for the neutrophil reaction of the three donors towards isolate 2010–1718 at the bacterial concentrations 0.75, 7.5 and 75 bacteria/neutrophil cell were 8.0 (range 7.9-11.0), 9.7 (range 9.4-17.5), and 12.6 (range 12.4-19.6) (Table 1). The three donors had similar starting points (7737, 8405 and 8551 middle fluorescence intensity), but one donor responded considerably more than the other two when *C. concisus* was added (Table 2). This could be caused by inter-personnel difference in the CD11b up-regulation capability or possibly that the more responsive donor has previously been exposed to *C. concisus.*

**Table 1:** **Dose-dependency of neutrophil up-regulation of CD11b at the given ratio of *C. concisus *to neutrophils compared to the expression when cells were exposed to PBS**

**Isolate**	**Ratio 0.75:1**	**Ratio 7.5:1**	**Ratio 75:1**
1718	8.0	9.7	12.4
197540	6.7	9.2	13.5
ATCC	7.4	8.7	11.9
75775	7.1	8.9	12.7
119100	7.1	12.5	15.1
118452	8.4	11.9	18.0

Values are presented as median of three experiments.

**Table 2:** **Dose-dependency of up-regulation of CD11b in neutrophils from three different donors at the given ratio of *C. concisus *to neutrophils compared to the expression when cells were exposed to PBS**

**Donor 1**	**Ratio 0.75:1**	**Ratio 7.5:1**	**Ratio 75:1**
1718	8.0	9.4	12.6
197540	6.1	8.5	13.6
ATCC	7.4	8.7	11.9
75775	7.1	6.9	10.4
119100	6.1	9.1	15.1
118452	8.4	11.9	18.0
Donor 2			
1718	7.9	9.7	12.4
197540	7.0	9.2	12.6
ATCC	6.5	8.5	11.8
75775	6.1	8.9	12.7
119100	7.1	12.5	14.4
118452	7.7	9.8	12.8
Donor 3			
1718	11.0	17.5	19.6
197540	6,7	15.2	18.7
ATCC	9,9	13.9	18.1
75775	8.3	16.0	19.5
119100	7.7	16.5	19.0
118452	10.6	16.7	18.9

Values are presented as median of three experiments.

### Oxidative burst response of neutrophils opsonised with pooled *serum* from healthy donors

**Figure 1:**
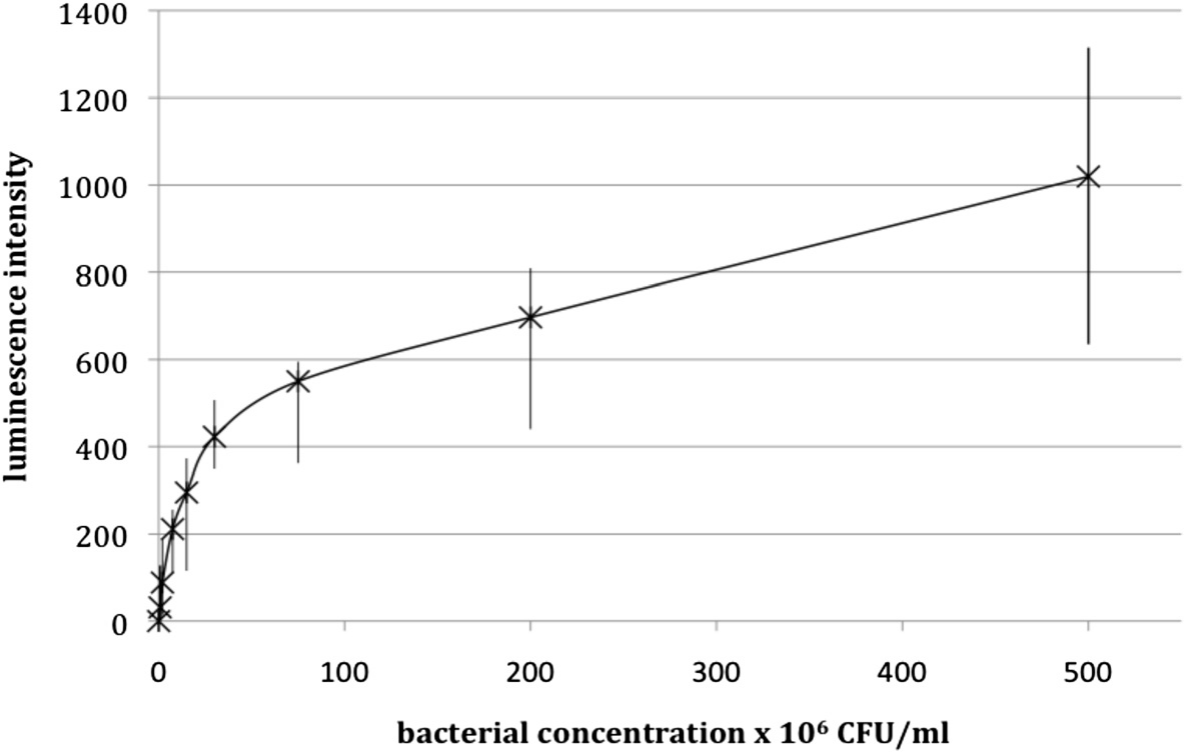
**Degree of luminescence intensity generated by the oxidative burst response of neutrophils from healthy adult donors towards *C. concisus *isolate 2010 *– *1718 opsonised with heat-treated pooled serum, in ratios ranging from 1:0.75 to 1:500 (neutrophils to bacteria).** Neutrophil concentration was held constant at 2×10^6^ cells/ml as the bacterial concentration was increased. Results are presented as mean values and ranges of 4 experiments performed in duplicates.

Measurement of the oxidative burst response of neutrophils from healthy donors towards *C. concisus* isolate 2010–1718 at concentrations between 0.75 and 500 CFU/neutrophil, using luminol dependent chemiluminiscence, expressed as ratios between the response towards *C. concisus* opsonised with pooled heat-treated serum and the response of unstimulated cells, showed a dose–response pattern (Figure 1). Values presented are median ratios of 4 experiments. This finding reflected that neutrophil cells did in fact react towards *C. concisus*, at first increasing the response rapidly as bacterial concentration increased, but as the concentration of *C. concisus* reached 75 CFU/neutrophil, the increase in response waned to a lower gradient that appeared to be constant.

**Figure 2:**
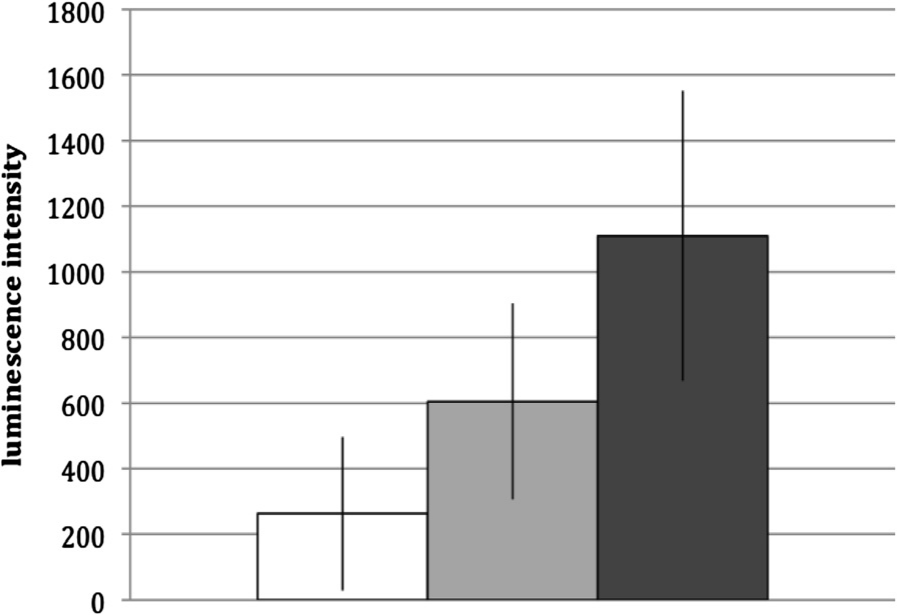
**Degree of luminescence intensity generated by the oxidative burst response of neutrophils from healthy adult donors towards *C. concisus *isolate 2010–1718 in the ratio 1:75 (neutrophils to bacteria) opsonised with heat-treated pooled serum (dark grey), compared to the response without serum (light grey) and the response when no bacteria but only neutrophils and serum are present (white).** Results represent mean values ± 2 standard deviations of 54 experiments performed in duplicates.

Measurements of oxidative burst response were carried out for neutrophils from 54 healthy donors exposed to *C. concisus* isolate 2010–1718 opsonised with pooled heat-treated serum as well as *C. concisus* alone and compared with baseline neutrophil response in PBS mixed with pooled heat-treated serum. The response of neutrophils was highest when *C. concisus* was opsonised with pooled heat-treated serum (median: 1110 luminescence intensity units, 95% CI: 667–1553 luminescence intensity units). Neutrophil cells exposed to *C. concisus* without opsonisation in serum also react (median: 605 luminescence intensity units, 95% CI: 306–904 luminescence intensity units, p<0,001), but less aggressively than when bacteria are opsonised with serum (p<0,001) (Figure 2) demonstrating the contribution of immunoglobulin dependent opsonic capacity in the neutrophil response to *C. concisus*.

### Neutrophil reaction towards different strains of *C. concisus*

Comparing the neutrophil reactions towards six different isolates of *C. concisus* on different steps of the inflammatory response demonstrated a similar pattern of response towards all isolates. The median ratios of CD11b up-regulation towards bacteria at a concentration of 75 CFU/neutrophil compared to the reaction of unstimulated cells in the range from 11.9 to 18.0, and the median ratios of oxidative burst response of neutrophils towards the same amount of bacteria compared to the reaction of unstimulated cells ranges from 2.4 to 3.2 (Table 3).

**Table 3:** **Neutrophil activation by different isolates of *Campylobacter concisus***

**Strain**	**CD11b up-regulation**^**1 **^**(n=3)**	**Oxidative burst response**^**2 **^**(n=4)**	**Sex/age**	**Clinical characteristic**
2010-1718	12.6 (12.4-19.6)	3.0 (2.7-3.9)	F/2	Bloody diarrhoea
2012-197540	13.5 (12.6-18.7)	2.5 (2.1-2.9)	F/58	Diarrhoea
2009-75775	12.7 (10.4-19.5)	2.4 (2.1-3.6)	M/29	Crohn’s disease
2009-119100	15.1 (14.4-19.0)	2.7 (2.0-3.3)	F/63	Collagenous colitis
2009-118452	18.0 (12.8-18.9)	3.2 (2.9-4.1)	F/19	Crohn’s disease
ATCC 33237	11.9 (11.8-18.1)	2.8 (2.5-3.0)		Gingival sulcus

^1^Ratio between the expression of CD11b on the neutrophil cell surface after exposure to *C. concisus* compared to the expression when exposed to PBS. ^2^Ratio between the neutrophil oxidative burst response when exposed to *C. concisus* compared to the oxidative burst response when exposed to PBS. Values are presented as mean (range).

### Oxidative burst response of neutrophils to *C. concisus* opsonised with patient *sera*

When measuring the oxidative burst reaction of neutrophils towards *C. concisus* opsonised with heat treated serum from 40 C*. concisus* infected patients they did not generate a larger response than healthy adult donors (data not shown). Furthermore, the opsonic activity in heat treated serum from patients with *C. concisus* infection remained unchanged four weeks after the baseline visit (p=0.15). The opsonic activity of heat treated serum from patients had no association with clinical characteristics (i.e. fever, bloody stools, duration of illness, or age of the patient).

## Discussion

*Campylobacter concisus*, originally associated with the human oral cavity, has been proposed as an emerging pathogen causing gastrointestinal disease in humans [13]. *C. concisus* requires specific hydrogen-enriched microaerobic conditions for growth, probably causing it to be underreported, since such conditions are not frequently used in routine clinical laboratories. Furthermore, the isolation from human faecal specimens is facilitated by the filter method [14], which may not be in general use in diagnostic laboratories. To investigate the potential of this organism to initiate inflammatory disease, we examined the pathogen-host relationship with use of neutrophil cells of healthy donors. Firstly the expression of CD11b on the surface of phagocytes exposed to *C. concisus* was measured using flow cytometry. CD11b is necessary for the adhesion of neutrophils to the vascular endothelium in response to cytokines released from tissue invaded by a pathogen. Up-regulation of CD11b on the surface is one of the first signs of phagocytes responding to a pathogen. Secondly, measurements of oxidative burst response were carried out both towards *C. concisus* opsonised with heat-treated serum from diarrhoeic *C. concisus* infected patients, *C. concisus* opsonised with heat-treated pooled serum from healthy donors and *C. concisus* alone.

Clinically*, C. concisus* have recently been associated with prolonged diarrhoea in 80% of patients with culture-positive *C. concisus* gastroenteritis, whereas the pathogen gives rise to an elevated C-reactive protein level only about half as often as *C. jejuni/coli* (48% compared to 94%) [6]. These findings could be consistent with an evasive property in *C. concisus* towards the immune system, causing the responsiveness of neutrophils to be compromised. The observation also suggests that the pathogenesis of *C. concisus* associated pathology differs from the acute mucosal gastroenteritis well described for *C. jejuni/coli*. A more superficial mucosal interaction with *C. concisus* is likely, and consequently less involvement of a systemic inflammatory response (C-reactive protein increase) is to be expected. In this study we found that neutrophil cells from healthy donors respond to *C. concisus* in a dose-dependent manner both when investigating the expression of CD11b on the cell surface and the outburst of reactive oxygen intermediates (ROIs). The extracellular release of toxic oxygen radicals from neutrophils in the acute inflammation might contribute to the local tissue damage during infection and potentiate the inflammatory reaction. Furthermore we showed that although neutrophils respond towards *C. concisus* without these being opsonised with serum the response is increased when bacteria are opsonised. Previous studies have found that opsonised *C. jejuni* generates a higher degree of neutrophil granulocyte response than non-opsonised bacteria [1,2,15] correlating well with our findings. Our study design does not allow us to definitely conclude whether opsonisation induces increased phagocytosis of *C. concisus*. Previous investigations of *C. jejuni* and *H. pylori*[2-4] have shown that opsonisation significantly increases phagocytosis in both complement-dependent and –independent assays. Combination of heat-treated and untreated serum produced a higher opsonic activity for *C. jejuni* stimulation of neutrophils [4].

Our findings did not indicate any major differences in the neutrophil stimulating properties of the six different *C. concisus* isolates investigated differing from the results investigating isolates of *C. jejuni* [3], where large variations was reported. In other assays of inflammatory mediators, however, heterogeneity of *C concisus* isolates has been reported [11]. It is possible that various subgroups of *C. concisus* exist and preliminary genetic data suggest *C concisus* to be a very heterogeneous species. However, for the five clinical isolates and the oral reference strain we observed no substantial difference in the responses upon neutrophil stimulation. It remains to be further examined if differences in genetic characteristics are translated into differences in the activation of the innate immune system of neutrophils. Moreover, it is striking that an oral isolate exhibited the same activity as the faecal isolates. At this point it is controversial if oral and faecal strains of *C. concisus* are linked or are separate subgroups. Several of the isolates investigated (75775, 118452, 119100 and 1718) have in a previous study been shown to have the ability to perturb the barrier function of HT-29/B6 intestinal epithelial cells [10]. Furthermore, the pathogenic potential of *C. concisus* has been demonstrated in models of cellular invasiveness in human intestinal epithelial cells [16]. Whether or not *C. concisus* is able to be invasive in the host is unknown, but no report on bloodstream isolation of *C. concisus* has been published. *C. concisus* opsonised with serum from *C. concisus* positive patients did not generate a larger neutrophil response than those opsonised with pooled serum from healthy individuals, indicating a weak IgG response towards the bacteria. However, we cannot exclude that the sensitivity of the assay employed in the present study does not allow to detect specific IgG activity. Previously, another mucosal gastroenteric pathogen, *Helicobacter pylori*, was reported to be opsonised equally well with sera from infected patients and healthy controls [17]. So far, no assay of serum immunoglobulin concentration specifically for *C. concisus* has been reported, so the magnitude of systemic antibody response is unclear.

## Conclusions

Human neutrophil cells show an innate immune reaction towards *C. concisus* even at low concentrations at both the initiation of the immune-inflammatory response with up-regulation of the adherence molecule CD11b as well as later in the response with the outburst of reactive oxygen radicals. The latter increases when the pathogen is opsonised with serum, but we did not observe immunoglobulin associated amplification of the response in heat treated serum from patients with *C. concisus* infection. The findings of pro-inflammatory activation in neutrophils support the potential and clinical relevance of *C. concisus* as an emerging enteric pathogen. The outcome of *C. concisus* associated diarrhoea is very diverse and it remains to be investigated if the responses of the innate immune system correlate with the clinical presentation.

## Methods

### Isolates of *campylobacter concisus*

Five faecal isolates from diarrheic patients were selected for the study (*C. concisus* isolates 2010–1718, 2009–75775, 2009–118452, 2009–119100, and 2012–197540 as previously published [18]) as well as the oral reference strain *C. concisus* ATCC 33237). Isolates were cultured on 5% horse blood agar, containing 1% yeast extract (SSI Diagnostica, Hillerød, Denmark), and incubated at 37°C in a microaerobic atmosphere with 3% hydrogen. Final confirmation was obtained through a species-specific real-time PCR based on the cpn60 gene, as described by Chaban et al. [19]. After two days bacterial cells were suspended in phosphate buffered saline (PBS) to a concentration of approximately 75 × 10^6^ CFU/ml (absorbance 0.25 McFarland units) and transferred to 2 ml aliquots that were stored at −20°C until use.

### Measurement of optical density

To ensure that the concentration of the six different *C. concisus* isolates were the same throughout the experiments, measurements of optical density were carried out using Spectramax (Molecular Devices, LLC, USA) at a wavelength of 600 nm and converted to concentration using a linear model made from a series of measurements of the isolate 2010–1718.

### Isolation of neutrophil granulocytes

Peripheral venous blood was taken from healthy adult donors, anticoagulated by EDTA and separated by centrifugation (500 g for 30 minutes) on polymorphprep (Axis-Shield, Dundee, Scotland). Neutrophils were washed three times in PBS-AG (100 ml PBS, 0.1g albumin, 0.2 ml 50%glucose), and the final neutrophil cell concentration was adjusted to 2 × 10^6^ cells/ml suspended in PBS-AG using sysmex xs-1000i (Sysmex Corporation, Kobe, Japan).

### Serum samples

Serum samples were taken from 40 diarrheic patients with culture-positive *C. concisus* stool samples approximately 4 weeks after the diagnosis had been made [6]. For the pooled serum used for controls, serum samples from five healthy male donors were used. Serum was transferred to cryovials and stored at −80°C until use. Before use serum samples were heat treated at 56°C for 30 minutes to inactivate the complement system and then diluted to 1:200 with PBS. In prior experiments this concentration was found to be optimal for the oxidative burst response assay (data not shown).

### Adherence molecule expression assay

**Figure 3:**
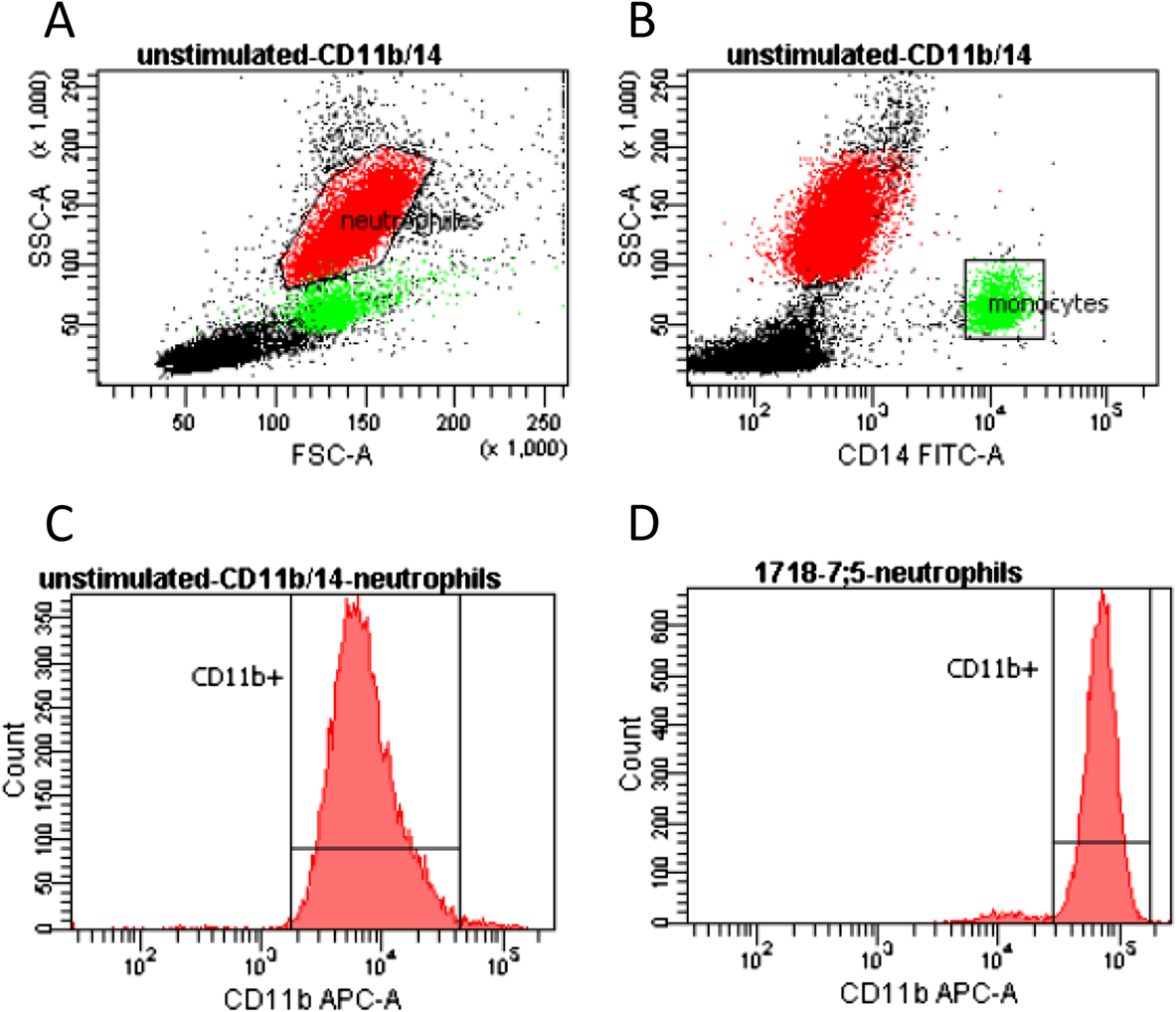
**Forward-side scatter dot plot of unstimulated neutrophils (A), side scatter dot plot with anti-CD14 identification of monocytes (B) as well as histograms of CD11b up-regulation in unstimulated (C) and stimulated (D) neutrophils stimulated with *C. concisus *isolate 2010–1718 at the ratio 7.5:1.**

Flow cytometry was used to quantify the expression of CD11b on neutrophils exposed to six different isolates of *C. concisus*. Each isolate of *C. concisus* was diluted to concentrations of 0.75, 7.5 and 75 times the average concentration of neutrophils (5x10^6^/ml) before being added to heparinised whole blood from a healthy adult donor in the ratio 1:1. The samples were incubated for 30 minutes at 37°C. Unstimulated controls were run in parallel. Immediately after incubation the samples were cooled on ice and 10 μl of anti-CD11b (APC-marked, Becton, Dickinson and Co., New Jersey, USA) and anti-CD14 (Fitc marked; Dako, Glostrup, Denmark) antibodies were added. Anti-CD14 antibodies were used to identify the monocyte population in the samples. To one sample of each isolate 10 μl of isotype control for Fitc and APC (isotype Fitc/APC/PE antibodies, Dako) were added. After an incubation time of 45 minutes at 5°C erythrocytes were lysed using lysing solution (Becton, Dickinson and Co.), the leukocytes were washed in PBS and suspended in sheath fluid (Becton, Dickinson and Co.) [20,21] and the samples run using a FACScanto fluorescence flow cytometer (Becton, Dickinson and Co.). The results were analysed using FACSDiva software (Becton, Dickinson and Co.). A forward/side scatter dot plot correlated with a forward scatter/anti-CD14 dot plot was used to identify neutrophils and exclude monocytes (Figure 3). The expression of CD11b was analysed using a histogram of the region containing neutrophils. Results are presented as a ratio between mean fluorescence intensity and the mean value of unstimulated cells.

### Oxidative burst response assay

Luminol dependent chemiluminiscence was used to measure the oxidative burst response of neutrophils against *C. concisus* isolate 2010–1718 opsonised with heat treated patient serum and heat treated pooled donor serum, respectively. For each serum sample a negative control with PBS was run in parallel. All samples were run as duplicates and on every plate a control without serum was run in parallel. Forty patient samples were run three times each with 2–3 patient samples per plate. Each plate contained a control with pooled serum and neutrophils from a healthy donor. In total 54 plates were run. 100 μl luminol, 25 μl heat-treated serum, 50 μl neutrophil cells (2 × 10^6^ cells/ml) and 100 μl bacterial suspension or PBS was added to the wells of a white polysorb microtiter plate and run immediately in at 37°C using Lumistar (BMG Labtech, Ortenberg, Germany). The peak value was reached after approximately 30minutes and each experiment ran for at least 40 minutes. Peak luminescence values were corrected by subtracting the peak value of unstimulated cells.

When comparing the neutrophil reaction towards different *C. concisus* isolates the test was run simultaneously for all strains with neutrophils from the same donor and heat-treated pooled serum using the same quantities as mentioned above. All samples were run in duplicates. The results are presented as ratios between the peak luminescence value and the value of unstimulated cells.

### Statistical analysis

Statistical analysis was carried out using probit plots and the student’s t-test for normally distributed data and the Wilcoxon-test for non-normally distributed data.

### Ethical approval

Scientific and ethics approval for the study was obtained from the Ethics Committee for North Denmark Region (N-20080056).

## Competing interests

The authors declared that they have no competing interests.

## Authors’ contributions

NBS performed the experiments and the first draft of the manuscript, HLN provided clinical samples and patient data, KV supervised the experiments, and HN designed the study, supervised the experiments and contributed to the manuscript. All authors read and approved the final manuscript.
